# Assessing stigma: Health and social worker regard towards working with people using illicit drugs in Athens, Greece

**DOI:** 10.1186/s12954-024-01091-x

**Published:** 2024-09-26

**Authors:** Cristina Temenos, Aliki Koutlou, Sotiria Kyriakidou, Sofia Galanaki

**Affiliations:** 1 University of Manchester, Manchester, UK; 2 Psychiatry Department, “ATTIKON” University General Hospital, Athens, Greece

**Keywords:** Stigma, People who use drugs, Healthcare professionals, Social work professionals, Medical Condition Regard Scale, Training, Supervision, Burn-out, Greece, Athens

## Abstract

**Background:**

After the 2008 Global Financial Crisis and resulting economic austerity, the rise in illicit drug use engendered an increased need for people who use drugs (PWUD) to access medical care, compounded by the COVID-19 pandemic. Research shows that perceptions of medical staff towards PWUD facilitate or act as a barrier to accessing health care. This study provides a better understanding of health and social work professionals’ perceptions by assessing stigma levels towards PWUD in Athens, Greece.

**Methods:**

This is a mixed-method study. It calculates the stigma score for professionals (*n* = 60) and the stigma score associated with specific drugs based on the Medical Condition Regard Scale through a quantitative analysis of responses to a semi-structured online survey about attitudes of health and social work professionals towards PWUD. It draws on the qualitative analysis of 12 semi-structured interviews with 16 service managers, providers, and health services advocates working in the charity sector to determine whether perceptions of PWUD affect writing and implementing policy and protocols for services.

**Results:**

Stigma towards PWUD exists amongst health and social work professionals in Athens. Professionals who have worked with PWUD for longer periods of time, professionals who have had specific training on working with PWUD, and professionals who feel that they have the necessary training to work with PWUD all demonstrated a higher stigma score than those reporting the opposite. Cannabis and opioids were associated with lower stigma scores while shisha had the highest level of stigma associated with it. Finally, professional environments are not conducive to alleviating stigma as the lack of training specific to stigma, the lack of professional supervision, and worker burn-out are key barriers faced by professionals in their everyday practice.

**Conclusions:**

Reducing and eliminating stigma towards PWUD among health and social workers requires immediate action. Measures to be taken include: introducing training programs focused on stigma towards PWUD to healthcare providers, social workers, lawyers, police, the media; increasing professional supervision on field work for health and social workers; introducing low barrier health care and specialist units. Peers and field-focused organisations should meaningfully participate in drug and alcohol policymaking, program development, and implementation.

## Background

Since 2008, many European cities have witnessed an increase in negative health effects due to the Global Financial Crisis and resulting economic austerity programs. With the post 2008 increases in illicit drug use and associated diseases and other ill health effects that come with illicit drug use, such as HIV and Hepatitis B (HBV) and Hepatitis C (HCV), also comes an increased need for people who use drugs (PWUD) to access medical care [1]. Understanding perceptions of medical staff towards PWUD is crucial as it can either facilitate or act as a barrier to accessing health care and treatment [2–4].

Research on attitudes towards PWUD demonstrates that stigma levels are high globally and exist among most groups, including medical professionals and social service workers who work with PWUD [5]. In their study introducing the Medical Condition Regard Scale (MCRS) as an instrument for measuring perceptions, Gilchrist et al. found that medical professionals working in primary care (PC), general psychiatry and specialist addictions services across 8 European countries held significantly lower regard for substance users than for patients with depression or diabetes, while regard for working with PWUD was significantly lower than regard for working with drinkers [6]. They also found that staff with fewer than 10 years’ experience showed higher regard to working with PWUD than those who had worked between 10 and 20 years in their profession [6]. In their systematic review of studies focusing on Oceania, the United States, Ireland, the United Kingdom and Canada, van Boekel et al. found that health professionals generally hold negative views towards PWUD and lack “adequate education, training and support structures in working with this patient group” [7]. Their findings in the countries of focus are supported by a large number of more recent studies [8–15].

Although there are many studies on perceptions of healthcare professionals towards PWUD in the English-speaking world, such studies are limited in peripheral European countries, which were hit particularly hard by austerity after the Global Financial Crisis. An exception is Armaos and Tsiboukli, who focus on Greece to explore the extent to which medical students have knowledge and understanding of drug use treatment in their education [16]. In another study, Andreu et al. explore PC providers’ views towards substance use disorder (SUD) patients, the relationship between PC and addiction settings, and the perceived needs of PC providers regarding SUD patients in Spain [17]. The studies contextualise stigma towards PWUD in healthcare systems, demonstrating their differences and similarities. For instance, the Spanish healthcare system differs from the Greek system in its operation as in Greece primary care is not yet fully based on a GP network, though legislation has been put in place to change this and came into effect in 2023. Yet, they are both public, state-funded and there is free access to healthcare. Research has shown that stigma towards PWUD is common, but plays out in different ways, it is geographically uneven across countries and within cities [18–20]. Therefore, locally specific data is required to understand whether stigma exists amongst health and social service providers who work with PWUD. It is, thus, important to unearth the ways in which fiscal austerity across these countries affected perceptions of healthcare professionals towards PWUD.

Equally crucial is the impact of the public health crisis of COVID-19 on such perceptions. For instance, Dunlop et al. note that the presence of stigma among medical professionals was a barrier to care access for PWUD during the COVID-19 lockdowns, and this may have long-term effects on their overall health and wellbeing [21]. In a study on the lived experience of stigma among PWUD, participants noted that interactions with health professionals such as PC providers, pharmacists, first responders, and hospital staff discouraged people from accessing health services [22].

Against this background, the present study seeks to fill two gaps in the literature. First, it assesses the level of stigma towards PWUD amongst health and social work professionals in the understudied case of Greece. Second, in so doing, it seeks to provide a better understanding of the impact of different crises on the perceptions of professionals towards PWUD. Some further contextualisation is, however, necessary to fully appreciate the contributions of this study.

### PWUD and access to healthcare in Greece

Specialised care towards PWUD in Greece is provided by public bodies or corporate bodies under private law, almost all of which receive full or partial government funding [23]. These bodies include public hospitals, self-governing organisations under the supervision of the Ministry of Health, non-governmental organisations and private clinics. Healthcare for substance use disorders addresses physical and mental health, with treatment administered on an outpatient services basis. The main forms of outpatient treatment involve psychosocial interventions and opioid substitution treatment (OST) offered through specialised drug treatment and counselling centres. OST is the most frequently offered treatment and only the Organisation Against Drugs (OKANA) has legal permission to establish, operate and monitor OST programmes. It is available across most cities in Greece and the substances used in it are methadone and buprenorphine. Inpatient treatment is also available. It is provided by residential drug treatment units, therapeutic communities, and prison units through a 21-day treatment programme. In most of these units, psychosocial treatment, mental health care, case management and referral to medical and/or social services are available.

Although data availability on substance use at national and European level is limited, the available data suggest that the role of primary healthcare and healthcare professionals in detecting and addressing drug related problems is narrow. Specifically, 12,480 people received drug treatment in Greece in 2017, with 9 out of 10 being treated in outpatient settings, and, specifically, in OST programmes [23]. Since the unravelling of the Greek debt crisis a decade ago, there has been a surge in: drug use, the number of people receiving OST treatment, and the use of synthetic drugs, such as shisha, becoming more prevalent among PWUD [24, 25].

In Greece most PWUD end up receiving treatment largely after self-referrals. Based on the latest available data by the European Union based statistical and research centre for substance use, European Monitoring Centre for Drugs and Drug Addiction (EMCDDA), there were 75.9% self-referrals and referrals from friends and family, 3.9% primary care GP referrals, 6.1% referrals from drug treatment centres and 3.9% referrals from other health, medical or social service in 2021 [26]. This highlights the limited role that primary healthcare and healthcare professionals play in detecting and addressing drug related problems in the Greek healthcare system.

In their most recent report on Drug-related hospital emergency presentations in Europe, the EMCDDA does not report data for Greece [27]. It also remains unclear which health services (e.g. hospitals, A&E, GP services or mental health services) are in high demand by drug using individuals, nor which services they access with most frequency or their motivations in choosing which services to access. The evidence gathered is ad hoc and anecdotal.

To this end, the present study has two key objectives. First, it seeks to provide a better understanding of the perceptions of healthcare professionals towards PWUD by exploring the existence of possible discriminatory perceptions that undermine the access of PWUD to healthcare services. Second, it seeks to highlight the source of such perceptions such as lack of training or knowledge, burn-out, the barriers faced by healthcare professionals in their everyday practice. A motivating factor in this study was the World Health Organization’s 3AQ framework for the Right to Health, which mandates that health services be available, accessible, acceptable, and of sufficient quality. This research provides a step towards this goal in ensuring measurable, locally specific data to make research and training recommendations surrounding access to health care for PWUD, and in particular those experiencing multiple deprivations such as entrenched poverty, homelessness, and comorbidities.

## Methods

To assess the level of stigma towards PWUD amongst health and social work professionals in Athens, the study design used a mixed methods approach. It consisted of [1] a semi- structured online survey of the attitudes of health and social work professionals towards working with PWUD (see Appendix A, Survey) and the quantitative analysis of their responses; and [2] qualitative, semi-structured interviews with service managers, providers, and health services advocates working in the charity sector. Participation in both the survey and the interviews was limited to people who were currently working with or who had worked in the past in health or social work capacities with PWUD in Athens, Greece.

### Survey

The survey data were collected between November and December 2022. The survey was addressed to healthcare professionals working in the health care or social work sectors in Athens at the time or who have worked in these sectors in Athens in the past. Recruitment proceeded with the sharing the survey link via 53 professional listservs that included medical and social work professionals academics and students, through the social media sites Twitter (now X), Facebook, and LinkedIn, including posting the survey on 41 organisational Facebook pages or asking organisations to post it on their Facebook pages, as well as with email invitations to personal contacts of the research team. In all cases, the survey aim, the estimated time of the survey completion (10–15 min) as well as the use of non-personal identifiable information and anonymization of the data were communicated to potential participants. In addition, the survey itself included a brief but detailed outline of the aims of the research, information about how the research team was to store and process the data and offered contact details for potential participants to use in case they had questions or needed any clarifications. Finally, the survey was rolled out in Greek and English to ensure maximum participation by overcoming language barriers some professionals could have encountered.^1^ We translated the survey and all communications, including the study description, informed consent, email correspondence and survey advertisements for social media using the forward-backward method [2, 28].

### Survey measures

The first part of the survey asked participants for demographics information, including: sex (male, female), educational status, and employment status (part-time, full-time, self-employed, volunteering, unemployed). In addition to asking participants about their current position within the addiction field (doctors and nurses, addiction therapists, social workers, psychologists, or other), the survey asked for information related to their prior professional experience in the field, whether they were providing services to people who use drugs at the time they took the survey, their prior experience in service provision to people who use drugs, whether there are written or any other type of guidelines to protect PWUD from discrimination in the organizations they work, and to the total years/months of professional experience in the addiction field. Through offering accesses to such information, the study outlines the characteristics of the addiction field in Athens and of those working on it.

The second part of the survey explored the regard of health and social work professionals towards working with PWUD and, in turn, the existence of possible discriminatory perceptions that undermine the access of such patients to healthcare services. This part of our survey aimed to capture the score on the MCRS. The MCRS is an 11-item instrument, which was initially developed to capture ‘medical condition regard’, a construct which reflects “positive or negative biases, emotions, and expectations produced by medical condition descriptors” for medical students and caregivers by assessing the degree to which they found “patients with a given medical condition to be enjoyable, treatable, and worthy of medical resources” [29]. It is a valid and reliable instrument for exploring regard towards different medical conditions, with coefficient alpha = 0.87 and test–retest reliability = 0.84 [29]. The MCRS has previously been used in exploring the regard of healthcare professionals towards substance use disorders [2, 6] and that of students towards working with PWUD - for a systematic review see [30]. In this part of the survey, we used the 11 items of the MCRS (Appendix C, Figure C.1). Each item was measured on a 6-point Likert scale ranging from ‘strongly disagree’ to ‘strongly agree’. Six items (1, 2, 4, 6, 9, and 10) were scored with 1 = Strongly disagree and 6 = Strongly agree whereas five items (3, 5, 7, 8, and 11) were reverse scored with 6 = Strongly disagree and 1 = Strongly agree. Reverse scoring was mobilised for MCRS items using negative wording to minimise any effects from acquiescence responding. The maximum MCRS score is 66 reflecting highest regard, and the minimum score is 11 reflecting lowest regard among healthcare and social work professionals towards working with PWUD.

In this part of the survey, participants were also asked to best describe how they felt regarding the statements in terms of specific types of drugs by choosing an option among different types of drugs from a drop-down list. The options available included cannabis, opioids, shisha (a variant of crystal methamphetamines), cocaine, crack, MDMA and ecstasy, prescription drugs, benzodiazepines and other drugs/psychoactive substances. The statements used to determine these attitudes are in Appendix B (see Table B.2). In addition, this part of the survey examined the potential sources of low regard towards PWUD, as well as the barriers faced by healthcare professionals in their everyday practice that might inform their attitudes towards patients and towards specific drugs. The questions we used are listed in Appendix B (see Table B.3).

The final part of the survey included two open-ended questions which prompted participants to share their thoughts on (a) the biggest challenges that health care providers face in Greece in caring for PWUD; and (b) on any existing programs, treatments, or approaches that would help health care providers to provide more effective treatment/care for PWUD. Our aim when asking these questions was twofold. First, to gain a more in-depth understanding based on participants’ input on the source of stigma and discriminatory perceptions that undermine the access of PWUD to healthcare services. Secondly, to highlight the need for more targeted interventions and tools for addressing stigmatising and discriminatory perceptions of healthcare and social work professionals towards PWUD.

IBM SPSS Statistics 25.0 software was used to conduct the survey data analysis. We initially calculated descriptive statistics for the demographic, education, and employment information of our sample (Appendix B, Table B.1). The mean and standard deviation were calculated for the age and the total years or month of professional experience in the addiction field were calculated to have a more comprehensive understanding of those working in the healthcare and social work sectors in Athens. Descriptive statistics were also calculated for: (a) the items of the MCRS (see Appendix C, Figure C.1); (b) the statements and questions further dissecting the attitudes of professionals towards PWUD as well as the reasons behind them in the third part of the survey (Appendix C, Figures C. 1; C. 2 and 4; Appendix B, Table B. 3 ); (c) the items aiming to explore the attitudes of professionals in Athens towards specific types of drugs (Additional File: Appendix B, Table B.2).

Analysis included the calculation of the stigma score. Several variables of the dataset were used to quantify the stigma score for each participant. Out of the 16 statements and items that referred to stigma, the score of 8 of them had to be inverted.^2^ To test the for the reliability of the stigma score, we calculated the Cronbach’s alpha (α) coefficient [31]. The coefficient’s value was α = 0.167 when calculated for the intended set of 16 questions. Given the low reliability, the Cronbach’s Alpha coefficient was used repeatedly (Appendix B, Table B.6) and we proceeded with removing variables whose score was inverted to attain an acceptable value (α > 0.7). Indeed, the removal of the 8 variables led to α = 0.749 (Appendix B, Table B.6). A Cronbach’s Alpha value equal to 0.749 suggests that the eight variables used to measure stigma score are reasonably consistent with each other.

To further explore the level of stigma among professionals in Athens, we conducted parametric and non-parametric tests. To test for normality, we performed the Kolmogorov-Smirnov and the Shapiro-Wilk tests (see Additional file: Appendix B, Table B.8). In addition, as our data were ordinal, we conducted the Mann-Whitney (M-W), Kruskal Wallis (K-W), and Spearman tests to assess for statistical significance of factors contributing to stigma (see Table 1 in the Results section).

**Table 1:** Statistical significance of factors contributing to stigma

	Stigma Score		
	Asymp. Sig. (2-tailed)	Statistic/Coefficient	Df
Education (M-W)	0.612	204.000	-
Currently working in the addiction field (M-W)	0.296	193.500	-
Currently providing services to people who use drugs (M-W)	0.555	193.000	-
In your organization, are there written or any other type of guidelines to protect people who use drugs from discrimination? (M-W)	0.023	93.000	-
I believe I have the needed qualifications in order to effectively work with people who use drugs. (M-W)	0.000	4.500	-
I believe I have the needed qualifications in order to effectively address the challenges of my everyday job with people who use drugs. (M-W)	0.000	10.000	-
Is treating vulnerable populations such as people who use drugs included? (M-W)	0.060	136.500	-
Is treating vulnerable populations such as people who use drugs included? (M-W)	0.000	54.000	-
I would be interested to attend a training regarding treating people who use drugs. (M-W)	0.822	145.500	-
Has the economic crisis affected the quality of care provided to people who use drugs? (M-W)	0.157	92.000	-
Has the COVID pandemic affected the quality of care provided to people who use drugs? (M-W)	0.047	29.000	-
Are there specific programs, treatments, or approaches that would help health care providers provide more effective treatment/care for people who use drugs? (M-W)	0.001	35.500	-
Current position (K-W)	0.119	7.334	4
I feel especially compassionate toward patients who use this particular type of drug. (K-W)	0.848	0.329	2
Treating patients who use this particular type of drug is a waste of medical dollars. (K-W)	0.151	3.783	2
Patients who use the following type of drug are particularly difficult for me to work with. (K-W)	0.292	2.460	2
I enjoy giving extra time to patients who use this particular type of drug. (K-W)	0.008	9.719	2
I prefer not to work with patients who use this particular type of drug. (K-W)	0.648	0.869	2
Total years or months of professional experience in the addiction field (Spearman)	0.080	-0.283	58

### Interviews

Interview data sought to complement survey data. The initial research design was meant to conduct the qualitative interviews first, and to then launch the survey in early 2020. Research delays and then the onset of the COVID-19 pandemic meant that the survey launch was delayed until autumn 2022. Given the length of time between the initial interviews and the survey as well as a global pandemic, the research team elected to conduct follow up interviews with past participants to understand whether there were any changes over time in perceptions or practices. Interviewees were invited to take part in the survey; however, it is unknown whether they took the survey as no personal, identifiable information was collected for the surveys.

Overall, we conducted 12 semi-structured interviews with 16 health services managers, clinicians, and advocates working in the charity sector to assess their perceptions of stigma amongst medical professionals towards PWUD, and whether and if so how these perceptions affect the ways in which policy and protocols are written and implemented for services. Ten face-to-face interviews with 15 individuals were conducted between November 2018 and January 2019 while two follow-up face-to-face interviews with three people were conducted in December 2022. Initial interview participants were identified by contacting all public organisations that deliver drug treatment services, and by contacting advocacy organisations that work with PWUD. We reached out to initial participants in the follow up, however not all were reachable or available. All interview participants provided written informed consent prior to enrolment in the study. Furthermore, anonymity was promised to all participants and therefore we cannot list names, organisation names or job titles.

Interviews lasted between 45 and 60 min and were audio recorded and transcribed by authors 3 and 4 for analysis after receiving the informed consent of the interviewees. One initial interview was not audio recorded and subsequently excluded from coding analysis. Interview transcripts were inductively coded to dissect (i) the sources of stigma; (ii) the key limitations of and recommendations for the effective provision of care to PWUD; and (iii) for the follow up interviews, whether and how the landscapes of drug use and care for PWUD has changed during the Greek debt crisis and the COVID-19 pandemic [32, 33]. Reliability of our analysis was assessed through discussions within the research team (Authors 1, 2, & 3). This follows established qualitative analysis practices for public health research [34, 35]. The interviews were independently coded by authors 1 and 3 and then both coded again by author 1 to look for similarities and differences.

## Results

### Survey

The total number of professionals that participated in this survey is 60. The initial sample size was 115 individuals, however, 55 participants responded to less than 20% of the questionnaire and therefore the total number of valid responses is 60 individuals (see Appendix B, Table B.1). The majority of the survey respondents were women at 85% and 15% were men, with a mean age 40.7 years (standard deviation 12.22 years). The mean of the participant’s professional experience in the addiction field is 10.3 years (standard deviation 9.41 years). Educationally, 66.7% of participants had a Bachelors’ degree, and 33.3% had a Masters’ degree. The professions of the respondents were reported as doctors and nurses (23.7%), addiction therapists (18.6%), social workers (11.9%), psychologists (11.9%) and others (33.9%). Most of the participants reported that they were fully employed (75.9%) at the time that they completed the questionnaire. Each of other employment status identified, constituted less than 10.3% of the total sample i.e., part-time employment 6.9%, self-employed 10.3%, volunteering 5.2% and unemployed 1.7%. Furthermore, more than half of the participants (63.3%) were working in the addiction field at the time of the study, and 48.3% of the total sample had prior professional experience in the field of addictions. 65% of respondents reported that they were providing services to PWUD at the time of the survey, and 35% of the total sample did not have prior experience in service provision to PWUD.

Importantly, more than half of the participants (60.4%) stated that in their organization there were not guidelines, written guidelines or in any other form, to protect PWUD from discrimination. Based on two Likert scale questions in which 1 was “strongly disagree”, 2 “disagree”, 3 “not sure but probably disagree”, 4 “not sure but probably agree”, 5 “agree” and 6 “strongly agree” the respondents overall have showed a positive attitude towards people who use drugs (see Appendix C, Figures C.1. and C.2.). More specifically, the professionals have reported that they enjoy giving extra time to PWUD (4.27), they usually find something that helps patients who use drugs to feel better (4.18) and that they find working with PWUD to be satisfying (4.5). Moreover, they stated that they feel compassionate towards PWUD (4.6) and that they would not mind getting up on night calls to care for patients who use drugs (4.2) and, they support that insurance plans should cover PWUD to the same degree that they cover people with other conditions (5.42). Notably, the majority of the participants reported that they do not believe that caring for patients who use drugs is a waste of medical dollars^3^ (1.53). Finally, the respondents agree that more than half of PWUD are not motivated to enter treatment (4.32) and that they have unequal access to medical and social services compared to the general population (5.32%) (Appendix C, Figure C.2.).

The analysis showed that the participants feel especially compassionate towards patients who use opioids and cannabis (42.2% and 33.3% respectively), and that they enjoy giving extra time to them (40% and 35.6% respectively) (as shown at Additional file, Appendix B, Table B.2). On the contrary, as presented in Table 2 (see appendix), the respondents do not feel the same way for patients that use other type of drugs such as MDMA/ecstasy, prescription drugs, shisha, cocaine, benzodiazepines, crack or others. Moreover, a relatively high percentage of the respondents reported that they find it difficult to work with patients that use shisha (35.6%), they would prefer not to work with them (31.1%), and they consider that treatment of patients that use shisha is a waste of medical dollars (28.9%). Table B.3 in Appendix B highlights that nearly one quarter of respondents reported that they do not believe they have the needed qualifications to address the daily work challenges (25.6%) or to work in an effective way with PWUD (22.5%). Notably, the majority of participants reported that they would be interested in attending a training course regarding PWUD (79.1%) and a relatively high percentage of the respondents indicated that training should be required in order to work effectively with PWUD (22.5%). Moreover, the vast majority of participants agreed that both the economic crisis and the COVID-19 pandemic have affected the quality of care that is provided to PWUD (81% and 90.2% respectively) (Appendix B, Table B.3).

**Table 2:** Descriptive statistics and stigma score for having the needed qualifications to effectively address challenges

	*N*	Mean	Std. Deviation	Std. Error	95% Confidence Interval for Mean		Minimum	Maximum
					Lower Bound	Upper Bound		
No	10	4.1750	0.85025	0.26887	3.5668	4.7832	3.13	5.50
Yes	21	2.6786	0.44295	0.09666	2.4769	2.8802	2.00	3.38
Training required	6	2.9792	0.49634	0.20263	2.4583	3.5000	2.13	3.38
Other	2	3.1875	0.26517	0.18750	0.8051	5.5699	3.00	3.38
Total	39	3.1346	0.84409	0.13516	2.8610	3.4082	2.00	5.50

As shown in Table B.4 (see Appendix B) during their formal education, participants reported that they have primarily received: training related to mental health (25.2%) and patient communication training (18.1%). Trainings during formal education related to patient care, drug addiction, stigma and infectious diseases have relatively low percentage: 14.8%, 13.5%, 12.9%, and 12.3% respectively, while the training related to handling of drug paraphernalia was reported low at 3.2%. Post-graduation professional trainings mirror the frequency (see Appendix B, Table B.5), with the highest percentage receiving training for mental health and patient communication (21.2% and 16.5% respectively). The trainings that follow are the ones related to drug addiction (15.9%), infectious diseases (14.1%), patient care (14.1%), stigma (11.8%), while the training related to handling of drug paraphernalia still remains low (6.5%).

By using eight variables of the dataset we quantified the stigma score for each participant, as explained in the Methods section above. The values of stigma score are in the set of 1–6. The highest value it takes, the lowest stigma is recorded, while 3.5 is the “neutral” stigma. The mean of the stigma score that is 3.08 (Appendix B, Table B.7), demonstrates the presence of stigma among the participants in this survey.

As shown in Table 1 below, variables such as levels of education, current working position or the provision of service to people who use drugs were not found to affect the professionals’ stigma levels, however, there were several statistically significant findings. As shown in Table 3, participants that reported the availability of guidelines i.e., written or other type, in their organization, to protect people who use drugs from discrimination, have presented higher levels of stigma (2.7) compared to the ones that they reported who do not have any guidelines in their workplaces (3.3), and this was found to be statistically significant (*p* = 0.023).

**Table 3:** Descriptive statistics and stigma score for guidelines protecting from discrimination

	*N*	Mean	Std. Deviation	Std. Error	95% Confidence Interval for Mean		Minimum	Maximum
					Lower Bound	Upper Bound		
No	26	3.3615	0.91568	0.17958	2.9917	3.7314	2.13	5.50
Yes	13	2.7308	0.53240	0.14766	2.4090	3.0525	2.00	3.63
Total	39	3.1513	0.85550	0.13699	2.8740	3.4286	2.00	5.50

Participants that believe that they have the needed qualifications to effectively work with PWUD presented significantly (*p* = 0.000) increased stigma towards people who use drugs (2.6) in comparison to those who reported that training is required (3.08), while those who acknowledged that they lack in qualifications to work effectively with patients who use drugs presented no stigma towards PWUD (4.3) (see Table 4). Similarly, respondents who believe that they have the essential skillset and qualifications to address the challengers of the everyday work with PWUD in an effective way were found to present significantly (*p* = 0.000) higher stigma levels (2.6) than those that stated that training is required (2.9) or those that acknowledged that they do not have the necessary skillset for this purpose (4.1) (see Table 2). Furthermore, participants that had received training since their graduation that included vulnerable populations such as PWUD were more likely to present stigma (2.8) towards PWUD (*p* = 0.000), compared to those whose trainings since graduation did not include vulnerable populations (3.9) (see Table 5).

**Table 4:** Descriptive statistics and stigma score for having the needed qualifications to effectively work with PWUD

	*N*	Mean	Std. Deviation	Std. Error	95% Confidence Interval for Mean		Minimum	Maximum
					Lower Bound	Upper Bound		
No	9	4.2917	0.81250	0.27083	3.6671	4.9162	3.13	5.50
Yes	20	2.6563	0.44955	0.10052	2.4459	2.8666	2.00	3.63
Training required	9	3.0833	0.42848	0.14283	2.7540	3.4127	2.13	3.38
Other	2	3.1875	0.26517	0.18750	0.8051	5.5699	3.00	3.38
Total	40	3.1469	0.83680	0.13231	2.8793	3.4145	2.00	5.50

**Table 5:** Descriptive statistics and stigma score for inclusion of treating vulnerable populations as PWUD in guidelines

	*N*	Mean	Std. Deviation	Std. Error	95% Confidence Interval for Mean		Minimum	Maximum
					Lower Bound	Upper Bound		
No	12	3.9271	0.95116	0.27458	3.3227	4.5314	2.50	5.50
Yes	31	2.8065	0.50295	0.09033	2.6220	2.9909	2.00	3.75
Total	43	3.1192	0.82238	0.12541	2.8661	3.3723	2.00	5.50

The majority of participants reported that they believe that the COVID-19 pandemic has affected the quality of care provided to PWUD (90.2%) and the stigma levels among them is found to be present, yet relatively low (3.2). However, those who do not support the aforementioned statement (9.8%), were found to have higher levels of stigma (2.5), which is statistically significant (*p* = 0.047) (see Table 6).Another statistical important finding (*p* = 0.001) is that the professionals that have stated that there are not specific programs, treatments, or approaches that would help health care providers to provide more effective treatment for people who use drugs (26.2%), were less likely to show stigma towards PWUD (3.9) than those that stated that training is required (2.7), or those that reported the availability of such programs, treatments of approaches (2.8) (see Table 7).

**Table 6:** Descriptive statistics and stigma score for the impact of COVID on care provided to PWUD

	*N*	Mean	Std. Deviation	Std. Error	95% Confidence Interval for Mean		Minimum	Maximum
					Lower Bound	Upper Bound		
No	4	2.5000	0.27003	0.13502	2.0703	2.9297	2.13	2.75
Yes	37	3.2095	0.83388	0.13709	2.9314	3.4875	2.13	5.50
Total	41	3.1402	0.82263	0.12847	2.8806	3.3999	2.13	5.50

**Table 7:** Descriptive statistics and stigma score for available alternatives for providing more effective care to PWUD

	*N*	Mean	Std. Deviation	Std. Error	95% Confidence Interval for Mean		Minimum	Maximum
					Lower Bound	Upper Bound		
No	11	3.9886	1.01620	0.30640	3.3059	4.6713	2.13	5.50
Yes	21	2.8393	0.45094	0.09840	2.6340	3.0446	2.13	3.63
Training required	10	2.7875	0.55917	0.17683	2.3875	3.1875	2.00	3.38
Total	42	3.1280	0.83030	0.12812	2.8692	3.3867	2.00	5.50

Furthermore, professionals who reported that they enjoy giving extra time to patients who use opioids and cannabis, also showed as having the highest stigma scores (mean: 2.8, *p* = 0.008), followed by the ones that stated that they enjoy giving extra time to patients that use MDMA/ecstasy/shisha/cocaine/crack (3.4), while the ones related to benzodiazepines, prescription drugs or other drugs presented no stigma (3.7) (see Table 8 ).

**Table 8:** Descriptive statistics and stigma score for giving extra time to patients per type of drug

	*N*	Mean	Std. Deviation	Std. Error	95% Confidence Interval for Mean		Minimum	Maximum
					Lower Bound	Upper Bound		
MDMA/Ecstasy-Shisha-Cocaine-Crack	3	3.4583	0.26021	0.15023	2.8119	4.1047	3.25	3.75
Opioids-Cannabis	34	2.8904	0.73402	0.12588	2.6343	3.1466	2.00	5.00
Benzodiazepines-Prescription drugs - Other drugs	8	3.7656	0.94830	0.33527	2.9728	4.5584	2.75	5.50
Total	45	3.0839	0.82050	0.12231	2.8374	3.3304	2.00	5.50

### Interviews

Our interview sample consists of conducted 16 health services managers, clinicians, and advocates working in the charity sector. In many organisations, people had multiple roles, particularly those working for charities or advocacy organisations. Broadly, eight were service providers, two were advocates with lived experience of drug use, two were psychologists, two were social workers, two clinical directors, one doctor, and one legal representative. Overall, we found that interview responses backed up the survey findings as all interviews described stigma within the health system towards PWUD.

Emergency care, such as the emergency room/A&E and ambulance services were noted as particular sites where PWUD experienced stigma. One respondent noted that: “An ambulance coming to pick up a drug user… from Omonia Square, is a long shot. The ambulance will take too long to arrive. I know this from personal experience.” (Interview NGO Advocate 2, 2022). Another stated that when they call for an overdose, they are not able to say it’s the reason for the emergency: “They will never come. So, it’s always like ‘we have somebody unconscious and he’s dying.’ You have to know that they don’t pick up anymore when I call them from my phone. They don’t. I don’t know. Maybe, I am in the restricted list.” (Interview NGO Advocate 4, 2018). This suggests a primary barrier to the healthcare system for PWUD given that PWUD most often access health services through ambulance or emergency care.

Interviews also demonstrated that during times of crisis, in particular the Greek Debt Crisis of 2009, and the COVID-19 pandemic, PWUD’ access to services decreased. Interviewees gave several reasons for this. The primary ones included: lack of training, ingrained cultural bias towards PWUD, and a perception of PWUD as being difficult patients. Lack of training and uneven training on illicit drug use and the issues that PWUD face cannot be seen as a cause of stigma, however it is an aggravating factor in reasons why stigma towards PWUD remains entrenched in the medical system. Training can be seen as a preventative measure, and in public health, when there are efficiencies to be made, it is often prevention measures that are the first to be cut. Thus, an already uneven landscape of training was further reduced by severe austerity measures implemented throughout the Greek health system starting in 2008. As a respondent put it:

“When it comes to [health] services offered and targeted especially to drug users then stigma exists, but it’s much less compared to services offered to the general public amongst to which drug users. This was very obvious when the epidemic of HIV in 2011–2013 hit and drug users had to regularly use services of hospitals that were not so frequent about drug users in the past. The personnel were not trained to deal with the particularities, with the necessities, with the needs of this kind of population. And they [PWUD] were severely discriminated…everything happened very fast and in the middle of the [debt] crisis. There is a justification why the training didn’t take place. So, both sides were left, let’s say…not uncovered, but without the knowledge and the tools to deal with this co-existence.” (Interview NGO Advocate 3, 2018).

One drug service provider noted that even with formal training there are barriers to overcome due to culturally ingrained biases. “We have some formal training about stigma, but I think there is a hidden stigma inside the training if you know what I mean. ‘We must help and treat the poor people’, it’s kind of stigma. They are not in the same level with us [socially].” (Interview Drug Service Provider 1, 2018). This can also be due to a deservedness discourse, which is amplified when resources become scarce and decisions around who can access a resource, such health care, need to be made [36, 37]. In parallel, an NGO advocate noted that fiscal austerity in the country is contributing to stigma towards PWUD. As they explained:

“And of course, one of the biggest issues that we have is that health care providers …are in a sense discriminating with their behavior [towards] these patients. Since we are a country suffering from financial crisis and sometimes you can find many health care providers that …say we are not supposed to spend valuable resources –moneywise- resources that we have to treat people that probably will get re-infected, or they have moral defects and are not according to my perception how human beings should live. So, they discriminate them and they are not welcome.” (Interview NGO Advocate 1, 2018).

Deservingness of care is also associated with perceived difficulty in the patient: “And if you hear the doctors, you will hear a lot “he is a difficult patient” [PWUD] this is the most common you get from doctors. Especially from nurses as well. I don’t know if there is ever an education of staff for these things, how to handle people.” (Interview NGO Advocate 3, 2018). There was a general feeling that all medical professionals exhibited stigma towards PWUD, including those in the mental health and nursing fields.

Over time, little has changed with regards to stigma. In the four years between our initial interviews and the follow up interviews, they noted that nothing has really changed. This is despite the survey indicating more training had taken place. As a drug service provider put it: “Regarding the stigma, I don’t think anything has changed. It was like before. That is, there is a general stigma regarding drug addicts” (Interview, Drug Service Provider 3, 2022). Another advocate noted:

“I think that absolutely nothing has been done. Dealing with stigma is not that simple. Specific work needs to be done in some respects. One of these aspects could be to inform, raise awareness and train specific parts of the population such as security forces, mental health professionals and healthcare professionals in general, to educate the community on specific issues, empower the community… This has happened from time to time, sporadically.” (Interview NGO Advocate 2, 2022).

When asked why change was either non-existent or incremental, the key limitations of and recommendations for the effective provision of care to PWUD came down to resourcing for training as well as broader resourcing around overall workload. This was borne out by the survey results, which reported that there is a high level of burn out, not enough resources to perform the job well and not a high enough level of oversight or supervision. Recommendations included more consistent development of national training, as well as increasing specialised health services in the community, rather than hospital based as they are now.

## Discussion

This study examines the level of stigma towards PWUD amongst health and social work professionals in Athens, Greece and seeks to understand the role it plays in discrimination towards PWUD when accessing health services. We found that stigma towards PWUD exists amongst health and social work professionals in Athens, Greece. Both in the interviews and the survey, participants reported that PWUD have unequal access to health services as a result of stigma. This finding is in line with previous studies in other European countries and globally [5, 22, 38–40]. Another novel finding from the survey is that: people who have worked with PWUD for longer periods of time, people who have had specific training on working with PWUD, and people who feel that they have the necessary training to work with PWUD all have a higher stigma score than those reporting the opposite. These findings echo those of the influential study of Gilchrist et al., who found that staff with fewer than ten years’ experience showed higher regard to working with PWUD than those who had worked between 10 and 20 years in their profession [6]. Yet, we are not aware of any other studies that measure these aspects with regard to stigma. Further research is, thus, needed in a wider geographical context to understand how widespread this finding may be, and the causes behind it.

Our mixed-method study yielded two contradictory findings. On the one hand, our analysis of survey data found that those more confident working with PWUD presented higher stigma scores. On the other, interviews with advocates, including those with lived experience of illicit drug use suggested that PWUD feel more comfortable accessing specialist services. Interviews also show that, while service users still experienced discrimination based on stigma towards PWUD, it was felt by service users that the discrimination happened to a lesser degree. Yet, both interviews and the survey also showed a preference for more specialist services to help combat discrimination towards PWUDs in healthcare settings. The contradiction between higher stigma scores among those confident working with PWUD and the identified need by service users, advocates and providers to provide more specialist services suggests that the implementation of any such services should pay particular attention to raising awareness of stigma and its effects. It also requires providing up-to-date specialist anti-stigma training to service providers in these healthcare settings.

When breaking down attitudes towards people who used specific drugs, the survey found that perceptions of people who use MDMA/ecstasy, prescription drugs, shisha, cocaine, benzodiazepines, crack or other drugs had the highest levels of stigma associated with them. In contrast, cannabis and opioids had lower levels of stigma associated with them. Given that opioids are still one of the most common illicit group of drugs used in Athens, it was surprising that they ranked lower on the stigma score than other drugs. However, it was unsurprising that cannabis ranked lower on the stigma score than other drugs. Compared to users of other drugs, cannabis users in Greece have a relatively higher level of social acceptance [41].However, stigma towards cannabis users is still substantial in the country and Greece has some of the most punitive cannabis legislation in Europe. Furthermore, in a study of seven European countries, stigma towards cannabis users in Greece was higher than stigma towards cannabis users in other countries [41]. Shisha had the highest level of stigma associated with it, with medical professionals reporting their perception that it was a waste of medical dollars to treat people who use shisha. This could be due to the unpredictable and sometimes violent behaviour of people under the influence of shisha, it’s relative novelty as a substance in Greece and thus uncertainty as to how to treat people using shisha, as well the primary user group (rough sleepers) having existing stigma towards them [25]. Interviews also indicated a perceived lack of understanding of how best to treat shisha use in addiction treatment services, which could also contribute to the negative associations with people who use shisha.

A synthesis of the survey results and interview data reveals that professional environments are themselves barriers to alleviating stigma towards PWUD in three respects: training, supervision, and burnout. Participants noted that there is almost a complete lack of training specific to stigma, which is a key challenge for health and social work professionals in Athens in their everyday practice. Such training is offered by some drug service providers (Interview with Drug Service Provider 1, 2018; Interview with Drug Service Worker 1, 2018) and professionals are training themselves through experience and educating themselves on stigma (Interview NGO Advocate 2, 2022). Yet, existing training programmes, either overarching or specific to stigma, can also be stigmatising. As a participant put it:

“*[W]e have some formal training about stigma but I think there is a hidden stigma inside the training if you know what I mean. “We must help and treat the poor people” it’s kind of stigma. They are not in the same level with us. So… it will better if we think that we need to help the fellow people… that we might have the same problem today or tomorrow or yesterday…or our kid might have the same problem. It’s something equal…equalizing. So there’s some hidden stigma*,* you can see it in the language that it’s used…not like twenty years ago but we still use some expressions that are stigmatizing. Like…the French say “toxicomanes”…we have the same word here in Greece. It’s stigmatizing. In France it’s a formal term for this phenomenon. Like you say PWUD they say toxicomanes. But ‘manic’ in Greek means something bad*,* so we are trying to avoid it*,* but we still say it…because it’s written in the memory*,* and it gets out easily. The next generation might forget that word I hope” (Interview with Drug Service Provider 1*,* 2018).*

This further nuances our finding that people who have worked with PWUD for longer periods of time, people who have had specific training on working with PWUD, and people who feel that they have the necessary training to work with PWUD all have a higher stigma score than those reporting the opposite. Namely, the pervasiveness of stigma can further be associated with the quality of existing training programmes, i.e., how stigmatising and dated they are as no changes have been made from before the global financial crisis through to the context of the COVID-19 pandemic, a health crisis that put further stresses on an already under-resourced health system. People who see themselves as adequately trained are the ones with higher levels of stigma. Taken together with findings on lack of supervision discussed below, as well as high levels of burnout and resource restriction, focus or having the time to reflect on stigma is likely to receive lower or no prioritisation.

Furthermore, health and social work professionals in Athens noted the lack of supervision as a key challenge to their work both in the survey and in interviews. Not only do they work alongside of a more experienced colleague for a limited amount of time but they, instead of their employer, cover the costs for psychotherapeutic supervision (Interview with Drug Service Provider 2, 2018). The role of supervision in relation to alleviating stigma towards PWUD among mental professionals dealing with dual diagnoses has been previously highlighted in other contexts, such as in Australia [42] and in the UK [42, 43].

Our study demonstrates that supervision is a pertinent issue for all health and social work professionals, especially in the context of economic crises when healthcare is defunded, and the staff is reduced. It is also in this context that participants reported that worker burnout is another crucial challenge they face daily. As a participant explained, 10–15 years of working in the field is a fair amount of time to experience burnout (Interview with Drug Service Provider 1, 2018). This in line with studies of experiences of healthcare professionals as well as third-sector peer workers in the field in the US [44, 45] and Canada [46, 47].

Interview recommendations that increasing low-barrier and specialised health services in the community, rather than remaining hospital based were widely discussed. Low-barrier services for PWUD are normally seen as not having a requirement of abstinence to access the service. Specialised services are those that are specifically for PWUD. This can encompass anything from GPs to being able to receive opioid substitution therapy from pharmacies, to not being required to attend a specific hospital unit for all issues if a person who uses drugs is also HIV positive. Community based services would reduce the time burden on PWUDs and has a possibility of increasing access to stigma-free or stigma-reduced care, while also being a cost-effective measure for resource restricted health systems [48, 49].

This article has some limitations. First, interviews and the survey were conducted with people working in Athens, Greece, which may have shaped participant’s work experience. Athens is the largest city in Greece, and a capital city. Therefore, it has a wider health and social care infrastructure, more financial resources, and wider networks of people who have both worked with PWUD and who work in health advocacy organisations than other cities or towns in Greece. It is also likely that experiences of people working in urban settings differ significantly from those in rural settings outside the Athens metropolitan area. Athens however is a major global city within the European Union, and thus this analysis should be taken regionally, within the context of European cities. Second, while we initially had 115 survey responses, only 60 were valid. It is unclear why 55 participants responded to less than 20% of the survey. This could have been due to it taking longer than anticipated for some participants, internet reliability, or system error of the survey software. While we checked for the latter and did not find any error, this cannot be completely ruled out. The relatively high number of initial survey responses (115) demonstrates interest in the topic, and thus we can conclude that further research in this area in Athens and throughout Greece should be undertaken to gain a wider understanding of the situation. We also found interview findings mirrored the survey findings and therefore, while a limitation, the triangulation of these findings across two different methods contributes to our Finally, as noted in the Methods section, 10 semi-structured interviews with 15 individuals were undertaken with 2 follow up interviews with 3 individuals. Most potential interview participants and organisations we reached out to agreed to be interviewed in the first round of interviews, however there is always the possibility of self-selection bias in interview participants. We reached saturation on many of the themes discussed and note them as common perceptions among participants. It would have been useful to have followed up with all initial participants, yet not all were reachable. However, we do not think that these limitations significantly affected the analysis or findings from the data.

## Conclusions

In conclusion, we argue that the role of stigma towards PWUD needs to be taken seriously as a barrier to accessing healthcare. Based on findings, we have the following three recommendations: Training programs focused on stigma to healthcare providers, social workers, lawyers, police, the media; Low barrier health care units and specialist units; Peers and field focused organisations should meaningfully participate in drug and alcohol policy making, program development, and implementation. The development of targeted training programs focused on stigma, together with professional supervision, is an important tool for raising awareness of how stigma leads to discrimination of PWUDs in the entire cycle of accessing health services. It is also important to consider how trainings can be developed to target people at all career stages, including those who have a longer history of working with PWUD. The development of low barrier and specialist health units that are adequately staffed across the system is recommended, considering that specialist units also need to ensure the confidentiality of patients visiting the services. Finally, including peers and field focused organisations in the development and implementation of policy and services is an important factor in overcoming discrimination stemming from stigma towards PWUD.

## Data Availability

The datasets generated and/or analysed during the current study are not publicly available due to participant permissions and GDPR regulations. Survey data supporting the conclusions of this article is included within the article.

## Footnotes

1 Proficiency in English is a prerequisite for employment and very common among certain sectors of medical professionals, including doctors and therapists/psychologists. However, English language requirements are not the same for nurses, social workers or third-sector workers and, thus, their proficiency in English could not be assumed. It is also common in Athens for medical professionals from abroad to volunteer with street outreach organisations that provide health care to street-based PWUD, therefore the survey was offered in English as well as in Greek.

2 The eight statements that had to be inverted are the following: There is little I can do to help patients who use drugs; Patients who use drugs irritate me; Treating patients who use drugs is a waste of medical dollars; Patients who use drugs are particular difficult for me to work with; I prefer not to work with patients who use drugs; More than half of people with substance use disorder aren’t motivated to enter treatment; I have felt threatened during my encounters with patients who use drugs; I believe that people who use drugs do not have equal access to medical and social services compared to the general population.

3 The term “waste of medical dollars” is part of the validated survey tool used (the MCRS). Despite being American centric in its connotation, it was the wording used in the survey, therefore we have kept this terminology within the paper.

## Abbreviations

A&E: Accident and Emergency
EMCDDA: European Monitoring Centre for Drugs and Drug Addiction
GP: General Practitioner
MCRS: Medical Condition Regard Scale
OKANA: Organization Against Drugs
OST: Opioid Substitution Treatment
PC: Primary Care
PWUD: People who Use Drugs
SDG: Sustainable Development Goal
SUD: Substance Use Disorder
